# C-src Enriched Serum Microvesicles Are Generated in Malignant Plasma Cell Dyscrasia

**DOI:** 10.1371/journal.pone.0070811

**Published:** 2013-08-05

**Authors:** Giuseppe Di Noto, Lucia Paolini, Andrea Zendrini, Annalisa Radeghieri, Luigi Caimi, Doris Ricotta

**Affiliations:** Department of Molecular and Translational Medicine, Faculty of Medicine, University of Brescia, Brescia, Italy; INRS, Canada

## Abstract

Plasma cell dyscrasias are immunosecretory disorders that can lead to hematological malignancies such as Multiple Myeloma (MM). MM accounts for 15% of all hematologic cancers, and those diagnosed with MM typically become severely ill and have a low life expectancy. Monoclonal immunoglobulin Free Light Chains (FLC) are present in the serum and urine of many patients with plasma cell diseases. The biological differences between monoclonal FLCs, produced under malignant or benign dyscrasias, has not yet been characterized. In the present study, we show that endothelial and heart muscle cell lines internalize kappa and lambda FLCs. After internalization, FLCs are rerouted in the extracellular space via microvesicles and exosomes that can be re-internalized in contiguous cells. Only FLCs secreted from malignant B Lymphocytes were carried in Hsp70, annexin V, and c-src positive vesicles. In both MM and AL Amyloidosis patients we observed an increase in microvesicle and exosome production. Isolated serum vesicles from MM, AL Amyloidosis and monoclonal gammopathy of undetermined significance (MGUS) patients contained FLCs. Furthermore MM and AL amyloidosis vesicles were strongly positive for Hsp70, annexin V, and c-src compared to MGUS and control patients. These are the first data implying that FLCs reroute via microvesicles in the blood stream, and also suggest a potential novel mechanism of c-src activation in plasma cell dyscrasia.

## Introduction

Hematological malignancies may be a result of plasma cell dyscrasia such as MGUS, or smouldering multiple myeloma (SMM). MGUS can be diagnosed incidentally and can behave like a benign, asymptomatic entity, or it can progress (1% per year) to different hematologic malignancies such as multiple myeloma (MM) [1], [2]. The SMM asymptomatic plasma cell disorder carries a higher risk of progression (10% in the first 5 years) compared with MGUS [3]. Unfortunately, we currently lack reliable biological markers that allow us to predict which MGUS or SMM patient will progress to MM [4]. Improved detection techniques, at the molecular level, could help in disease management of patients diagnosed with MGUS, SMM, or MM.

B cell clones, either malignant or benign, normally produce high amounts of monoclonal immunoglobulins (paraproteins). Paraproteins are made up of intact immunoglobulins and either single light chains or, more rarely, single heavy chains. Kappa or lambda FLC have been long-considered a by-product of plasma cells. However, recently published data indicate that serum FLCs may account for some specific functions during immune response [5]. Bradwell and co-workers developed an assay that can detect only FLC in a milieu of free and bound LC [6]. The assay has now been widely adopted in clinical practice and has revealed a clear imbalance of the free kappa light chains versus free lambda light chains in plasma cell dyscrasia. In fact, an abnormal FLC ratio [7]–[9] has proven to be predictive for the progression of MGUS, solitary plasmacytoma of bone, amyloidosis, MM, Waldenstrom’s macroglobulinemia and SMM [10]–[12].

A subset of monoclonal FLCs possesses intrinsic pathogenicity, which is a trigger factor of diseases such as AL amyloidosis and light chain deposition disease (LCDD) [13], [14]. Amyloidogenic FLCs circulate through the vascular system and deposit as insoluble fibrils, which leads to kidney, heart, and lung damage. Renal damage is, in most cases, the earliest indication of systemic involvement [15], [16]. The cardiac involvement occurs in more than 50% of AL amyloidosis patients and is a leading cause of morbidity [17]. Paraproteins are thus able to induce different tissue damage. However, the molecular mechanism related to the clinical progression is not fully understood.

The first evidence of intracellular uptake of kappa and lambda FLC was published 10 years ago [18]. To date, only the trafficking pathways of FLCs in cardiac fibroblasts [19], and kidney epithelial and mesangial cells [20] have been studied, together with the function of FLCs in the tubulo-interstitial environment [21]. Receptor mediated endocytosis and metabolism of FLCs generate an intra-renal pro-inflammatory environment that exacerbates ongoing renal injury via src-Tyrosine Kinase (c-src), NF-kB activation, and nuclear translocation of p65, H_2_O_2_, and MCP-1 production. Not all FLCs are able to activate the proximal tubule; it has been hypothesized that this phenomenon involves the physicochemical composition of FLCs [21], [22]. The FLCs’ mediated generation of pro-inflammatory environment in the vascular bed has never been considered. But before acting in specific tissues, FLCs must cross the endothelial layer. Growing evidence suggests that microvesicles and exosomes play a major role in intercellular communication. Several studies demonstrate that exosomes can modulate immunoregulatory processes, set up tumour escape mechanisms, and transfer physiological information [23], [24].

In the present study, we characterize the internalization rate of different FLCs in endothelial and myocardial cells. We show that endothelial and heart muscle cell lines are both able to efficiently internalize kappa and lambda FLCs. After internalization, FLCs are rerouted in the extracellular space via microvesicles exosomes. We processed serum samples from MM, AL Amyloidosis, MGUS, and healthy patients as the control. We saw a significant increase in microvesicle exosome production only in MM and AL Amyloidosis patients. Furthermore, the serum vesicles containing FLCs from MM and AL Amyloidosis patients were strongly positive for Hsp70 and c-src compared to MGUS and control patients. Our data show a FLCs rerouting process via microvesicles and exosomes, and also suggest a potential novel mechanism of c-src activation and function. Finally, we show a new potential diagnostic target to evaluate the FLCs’ pathogenicity.

## Methods

### Ethics Statement

Serum samples from patients were collected from the Laboratory of Biochemical Chemistry, Azienda Ospedaliera Spedali Civili of Brescia (AOSCB). Waste serum samples, derived from routine analysis, were selected for diagnosis and paraprotein production, coded, anonymized and frozen at –80°C: monoclonal components were detected by high-resolution agarose gel electrophoresis/immunofixation (Interlab). Kappa/Lambda ratio of serum FLCs concentration was measured by particle enhanced nephelometry (Freelite assay, The Binding Site) on a Behring BNII Nephelometer (Dade Behring). The institutional review board of Azienda Ospedaliera Spedali Civili of Brescia approved the study in adherence with the Declaration of Helsinki. All patients provided their written consent to participate in this study. All traceable identifiers were removed before analysis to protect patient confidentiality, all samples were analyzed anonymously.

### Cell Culture and Treatment

- Cell Culture: HeLa (ATCC CCL-2) and H9C2 (ATTC CRL-1446) cells were grown in Dulbecco’s Modified Eagle’s Medium (DMEM) supplemented with 10% Fetal bovine serum (FBS) (Lonza), 1% Penicillin/Streptomycin (Lonza), 1% Glutamine (Lonza); HVEC [25] cells were grown in RPMI 1640 supplemented as was DMEM, at 37°C, 5% CO_2_.
- Starvation: cells were washed twice with PBS 1X (Lonza) and incubated with medium without FBS (starvation medium).
- Internalization assays: cells in starvation medium were incubated at 37°C with patients’ serum or not for different times as indicated in figures. The serum was diluted directly in the starvation media to the final FLCs concentration of 20 µg/mL. After serum incubation cells were washed carefully 3 times with PBS 1X at 4°C, they were incubated with 170U of trypsin (Lonza) 5 min at 4°C and 10 min at 37°C to remove any FLCs remained on the cells membrane. Cells were than harvested and centrifuged at 800×g 10 min. Pellet was re-suspended in 1 mL of PBS 1X and re-centrifuged. Cell pellets were re-suspended in extraction buffer (100 mM Tris, 150 nM NaCl, 1 mM EDTA) supplemented with protease inhibitor cocktail (P.I.) (32 mg/mL leupeptin, 6.4 mg/mL pepstatin, 40 mg/mL aprotinin; Sigma). The extracts were disrupted by sonication (Bandelin, Sonopuls) on ice for 20 seconds at 60% of maximum power, then centrifuged at 800 × g for 10 min at 4°C (Centrifuge 5417C, Eppendorf).

### Antibodies and Immunoblotting

The following antibodies were used in our experiments: sheep anti-lambda FLC, sheep anti-kappa FLC (The Binding site), mouse anti-clathrin heavy chain, mouse anti-LAMP-1 (BD Transduction Laboratories), mouse anti-α-tubulin (Millipore: Mab1637), DAPI (Invitrogen), rabbit anti c-src (Santa-Cruz), mouse anti-Annexin V (Santa-Cruz), mouse anti-Caveolin (Novus). Cell pellets for western blotting were treated as described above. The supernatants were normalized for protein concentration (Bradford Assay), boiled in reducing SDS sample buffer (80 mM Tris, pH 6.8, 2% SDS, 7.5% glycerol, 0.01% bromophenol blue) supplemented with 2% 2-mercaptoethanol (Sigma) for 5 min at 95°C and separated by SDS–PAGE on a acrylamide/bisacrylamide (12.5%) gel. To visualize FLCs signal we performed electrophoresis under native conditions: samples were processed as described earlier, resuspended in non-reducing sample buffer and boiled for 5 min at 95°C. Samples were run in a native gel (12.5% acrylamide–bisacrylamide without SDS) and analyzed by western blotting (WB).

Samples were transferred for 1 h onto PVDF membrane, blocked overnight with 5% fat-free milk, 0.05% Tween-20 in PBS 1X. The PVDF membrane was incubated with the antibodies described above for 2 h in PBS Tween 0.05% +1% fat-free milk. The membrane was washed 3× for 10 min with PBS Tween 0.05% and incubated for 1 h with one of the following secondary antibodies: donkey anti-sheep (Jackson Immuno Research), goat anti-mouse and goat anti-rabbit (Zymed), sheep anti-mouse (Amersham), goat anti-mouse Femto (Pierce), goat anti-rabbit Femto (Pierce). Blots were detected using Supersignal West Pico or Supersignal West Femto (Pierce) or ImmobilonWestern (Millipore). Images were acquired using a G:Box Chemi XT Imaging system (Syngene).

### Microvesicles Purification and Fractionation

Microvesicles were extracted as reported previously [26], [27]. Briefly, to obtain microvesicles from cells: cells in starvation medium were incubated at 37°C with patients’ serum. The serum was diluted in the starvation media to a final FLCs concentration of 20 µg/mL for all samples. After 4 h of FLCs internalization, cells were washed with PBS 1X, treated with trypsin 170 U 10 min at 4°C and left in fresh starvation medium. After 16 h starvation medium was collected, centrifuged at 800×g for 30 min to eliminate cell debris, then centrifuged at 16 000×g for 45 min and finally spun at 100 000×g for 2 h (Optima MAX Ultracentrifuge, rotor TLA-55 Beckman), to obtain microvesicles as pellets. The pellets were re-suspended in 25 µL PBS 1X supplemented with P.I., clarified through a 0.1 µm filter (Millipore) to reduce the number of contaminating large vesicles shed from the plasma membrane and boiled with reducing or not reducing sample buffer 5 min at 95°C. The supernatant was precipitated with 10% trichloracetic acid (TCA) (Sigma) for 6 h, centrifuged 10 min at 12 000×g, washed with CH_3_COONa in ethanol 20%, centrifuged 10 min at 12 000×g, re-suspended in reducing or not sample buffer and boiled 5 min at 95°C. Samples were electrophoresed and analysed by WB.

Alternatively, to obtain microvesicles from serum, 500 µL of patients’ serum were processed with serial centrifugations (800×g for 30 min, 16 000×g for 45 min, 100 000×g for 2 h) and the pellets were re-suspended in 50 µL PBS 1X supplemented with P.I. Reducing or not sample buffer was added and the samples were boiled 5 min at 95°C. Samples were electrophoresed in SDS-PAGE and native conditions and analysed by WB.

Crude microvesicle samples from patient’s serum were processed for further fractionation using a discontinuous sucrose gradient as previously described [28].

Samples were normalized for protein concentration (Bradford assay) when possible, in alternative equal volumes of each sample were loaded on a acrylamide–bisacrylamide gel.

### Scanning Electron Microscopy (SEM)

Exosomes were purified from 500 µL serum with serial centrifugations. Pellets were fixed with 2.5% glutaraldehyde (Sigma) in PBS 1X for 1 h. After washing twice with PBS, the fixed pellets were dehydrated with an ascending sequence of acetone (25%, 50%, 75%, 90%, 100%). Acetone was then washed away with high pressure liquid carbon dioxide (critical point dryer CO_2_, Balzers Union). Samples were analysed by SEM after gold sputtering (Balzers Union Sputtering System SCD 040**)**. In alternative serum pellets were re-suspended in 200 µL of PBS 1X and centrifuged 10′ with a Cytospin4 centrifuge (The Thermo Scientific) before CO_2_ dehydration and gold sputtering. All samples were visualized on a Philips 501 SEM operating at 15 kV.

### Immunofluorescences

HVEC cells were grown on glass coverslips of 35 mm of diameter until 60–80% confluency and incubated at 37°C with FLCs in patient’s serum (healthy patient (c), A1k, A2λ, MGUS and M1k, M2λ) or only with starvation medium for 4 h. The serum was diluted in the media to a final FLCs concentration of 20 µg/mL for all samples. Cells were fixed with 3% paraformaldehyde for 15 min, washed with NH_4_Cl for 15 min and permeabilized with 0.3% saponin in PBS 1X 3 times 10 min. Primary antibodies were incubated for 1 h and washed 3 times for 10 min with 0.3% saponin in PBS. Secondary antibodies were incubated for 45 min and washed as described above. Coverslips were mounted using an anti-fade mounting medium (ProLong Gold-Invitrogen) on a glass slide. Fluorescent microscopy was performed on a ZEISS Axiovert 100 microscope using the 63× Zeiss oil immersion objective. Images were processed with the use of Image pro-plus 4.5.1.

### Vesicles Quantification

After microvesicle purification from 500 µL of patients’ serum, pellets were re-suspended in 50 µL of extraction buffer (100 mM Tris, 150 nM NaCl, 1 mM EDTA) supplemented with P.I. and the protein concentration was determined using the Bradford assay.

### Lipid Composition

Microvesicle pellets from 1 mL serum (c and MGUS) and 500 µL serum (M1k, M2λ A1k and A2λ) were re-suspended in a total volume of 250 µL of chloroform/methanol (1∶1) (v/v), and lipids were extracted overnight at 40°C. The suspensions were then centrifuged for 10 min at 2000 × g, and the clear supernatants were dried with a dryer (Reacty Vap-Pierce). The residue was dissolved in a total volume of 100 µL chloroform/methanol (6∶4)(v/v). Each resulting solution was analysed by thin-layer chromatography (TLC). TLC was performed in a vertical development chamber (Camag, Muttenz, Switzerland) using 10 × 20 cm pre-coated TLC plates Silica Gel 60 (Merck) and a mixture of chloroform/methanol/water (65∶25∶4, by volume) as mobile phase. Lipids were visualized by spraying plates with 0.05% primulin solution dilute with acetone:water (8∶2 v/v).

### Vesicles lysis

500 µL of M2λ patient serum were processed for microvesicles purification with serial ultracentrifugation steps as described in Methods. The pellet (P1) obtained after the last ultracentrifugation at 100 000×g for 2 h was resuspended in 100 µL of trypsin for 30 min at 37°C and then ultracentrifuged at 100 000×g for 2 h. The pellet (P2) obtained was resuspended in 100 µL of extraction buffer +0.1% Triton X-100 (+) or not (−) for 30 min at 4°C and centrifuged at 100 000×g for 2 h. The pellet (P3) obtained was resuspended in 50 µL non reducing sample buffer, sonicated at 60% of maximum power and boiled at 95°C for 5 min. Supernatant from P3 (SN3) was precipitated by incubation with 10% trichloracetic acid (TCA) (Sigma) for 6 h, centrifuged 10 min at 12 000×g, washed with CH_3_COONa in ethanol 20%, centrifuged 10 min at 12 000×g, resuspended in 50 µL of non reducing sample buffer and boiled 5 min at 95°C. Input (0.25 µL total serum), P1 (2.5 µL), P3 (2.5 µL) and SN3 (2.5 µL) were loaded on a native gel and analysed by WB using anti FLC antibodies.

### Immunocapture Assay

Magnetic beads (PureProteome NHS FlexiBind Magnetic Beads, Millipore) were coupled with c-src or annexin V monoclonal antibody, as described in the PureProteome Magnetic Beads user guide. Discontinuous sucrose gradient fractions 6-7-8-9 were incubated with coupled magnetic beads over night at 4°C in gentle rotation. The beads were then placed on a magnet and the supernatant was discarded. The beads were washed 3 times with PBS 1X and finally resupended in 50 µL of reducing or non reducing sample buffer and heated at 95°C for 5 min. Samples were run in SDS-PAGE or native conditions and analysed by WB.

### Cytosol/Membrane Separation

HVEC cells in starvation medium were incubated for 4 h at 37°C with patients’ serum. The serum was diluted in the media to a final FLCs concentration of 20 µg/mL. After serum incubation cells were washed carefully 3 times with PBS 1X at 4°C, incubated with 170U of trypsin (Lonza) 5 min at 4°C and 10 min at 37°C or not. Cells were than harvested and centrifuged at 800×g for 10 min. The pellet was resuspended in extraction buffer supplemented with P.I., disrupted by sonication 60% of maximum power for 10 seconds and centrifuged at 800×g for 10 min (Centrifuge 5417C, Eppendorf). The homogenate was centrifuged at 100 000×g for 1 h at 4°C (Optima MAX Ultracentrifuge, rotor TLA-55 Beckman). The supernatant obtained was treated as cytosolic fraction, while the pellet (membrane fraction) was re-suspended in extraction buffer+P.I. The membrane fraction was solubilized by sonication at 15% for 10 seconds. All samples were re-suspended in reducing SDS sample buffer or non reducing sample buffer. The samples were heated at 95°C for 5 min and then electrophoresed in SDS-PAGE or native conditions and analysed by WB.

### Statistical Analysis

Significant differences among MGUS datasets and other samples were determined with Student’s *t*-test (Graph Pad). *P* values of less than 0.05 were considered statistically significant with * P<0.05, ** P<0.01 and *** P<0.001. Values were shown as mean ± Standard Error of the mean (SEM) of at least 3 experiments.

Significant differences among MGUS datasets and other samples in microvescicles protein content were determined with Mann-Whitney test (Graph Pad). * P<0.05, ** P<0.01 and *** P<0.001. Significant differences among controls (healthy donors) datasets and patients (MM, AL and MGUS) in FLCs pelletable fraction from serum were determined with Student’s *t*-test (Graph Pad) * P<0.05, ** P<0.01 and *** P<0.001. Data represents mean ± Standard Error of the mean (SEM) of 2 experiments.

## Results

### FLCs are Internalized in Endothelial and Heart Muscle Cell Lines

Studies show that dendritic cells, mesangial cells, kidney epithelial cells and cardiac fibroblasts internalize both kappa and lambda FLCs [18]–[20], [29]. FLCs are able to disrupt the normal physiology of organs such as the heart, peripheral nerves, lungs, and intestines. FLCs most commonly cause disruption in the kidney, a highly vascularized organ, both in MM and AL patients. We chose to investigate the FLC internalization rate of endothelial cells, cardiac myoblasts, and epithelial cells, because they serve as good models for the environment of the organ damaged by FLCs. Intracellular uptake and intracellular retention at different time points were performed with different paraproteins. We chose samples with high levels of kappa or lambda FLCs, derived from patients with Multiple Myeloma with AL Amyloidosis (named A1k and A2λ) or without (named M1k and M2λ). One MGUS patient with an IgGλ monoclonal component, in combination with monoclonal lambda FLCs, and two healthy donors (c) were also selected. All samples were classified for their paraprotein content as shown in Table 1. AL amyloidosis was excluded for M1k and M2 λ since periumbilical fat was negative at Congo red staining (Table 1).

**Table 1:** Description of the patients involved in the study.

Patient	Disease	K FLCs(mg/L)	λ FLCs(mg/L)	Ratio	S-IF	U-IF	Congo Red	IgA	IgG	IgM	OMB	Creatininmg/dL
A1k	AL Amyloidosis	1502	291,4	5,15	IgA/k	k	Pos.	48	541	35	20% plasma cells	0,85
A2λ	AL Amyloidosis	27	794,5	0,03	IgG/λ+ IgA/k	λ	Pos.	179	897	27	20% plasma cells	2,27
M1k	MM+renal failure	15000	8,04	1865	IgA/k	k	neg.	1970	438	10	90% plasma cells	9,25
M2λ	MM+renal failure	0,12	2750	0,00	λ	λ	neg.	25	357	10	20% plasma cells	7,2
MGUS	MGUS	9,73	1180	0,01	IgG/λ+λ	neg.	\	187	1471	89	n.a	0,7
C	Healty	12,5	18,1	0,69	neg.	neg.	\	253	1366	140	n.a	0,6
C	Healty	7,8	14,3	0,55	neg.	neg.	\	219	1140	119	n.a	0,55

S-IF: serum immunofixation; U-IF: urine immunofixation; Congo Red: periumbelical fat Congo red staining; OMB: Osteo-medullary biopsy; N.a.: not available.

**Figure 1:**
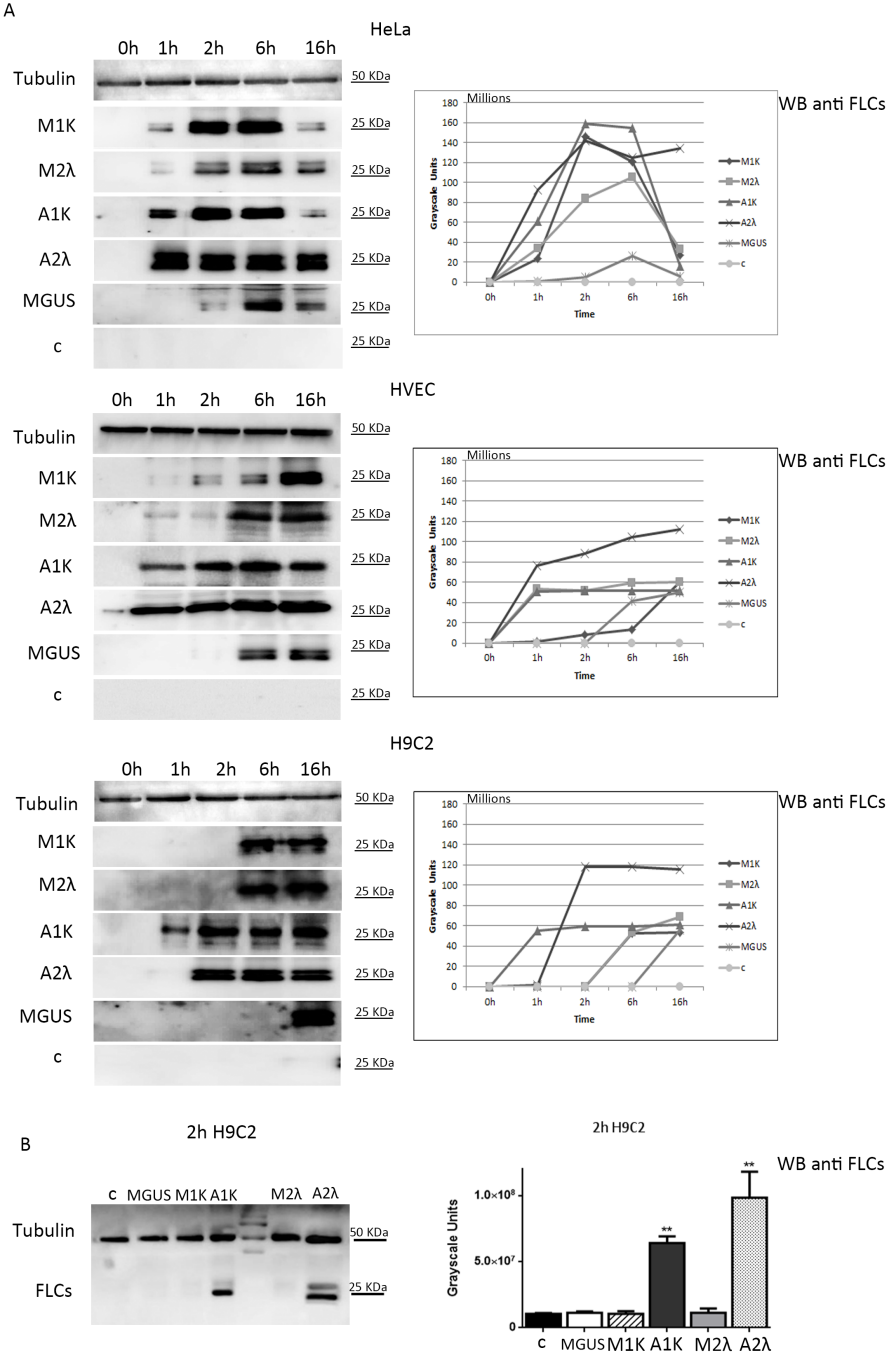
FLCs intracellular uptake. **(**A) 7×10^5^ HeLa, HVEC or H9C2 cells in starvation medium were incubated with serum containing FLCs from MM (M1k, M2λ), AL amyloidosis (A1k, A2λ) MGUS or healthy donor patients for 1, 2, 6 and 16 h at 37°C or not (0 h). The serum was diluted directly in starvation media to a final FLCs concentration of 20 µg/mL for all samples. After incubation cells were processed as described in “Methods”. Samples were re-suspended in non-reducing SDS-free sample buffer. 30 µg of each sample were run in a native 12.5% acrylamide–bisacrylamide gel. Western Blot (WB) analysis was performed with anti kappa, lambda FLCs and tubulin antibodies. Signals were quantified with the program Gene Tools. B) 7×10^5^ H9C2 cells in starvation medium were incubated for 2 h with serum diluted to a final concentration of 20 µg/mL of FLCs and treated as described in Methods to visualize the differences between AL, MM, and MGUS FLCs internalization rate. Healthy donor (c) was used as negative control. 30 µg of each sample were run in a native 12.5% acrylamide–bisacrylamide gel. WB anti FLCs and tubulin. The displayed values represent the mean ±SEM. Each fit was performed on a minimum of 2 separate experiments. ** P<0.01.

We incubated epithelial (HeLa), endothelial (HVEC) and cardiac (H9C2) cell lines with starvation medium containing the same final concentration of FLCs (20 µg/mL) at different time points. After incubation we treated the cell layers with 170U of trypsin 5 minutes at 4°C and 10 minutes at 37°C to eliminate FLCs that are still present on plasma membranes. This treatment was equally efficient in eliminating residual FLCs from the cell membranes for all tested serum samples (Figure S1A, B). Figure 1A shows that all cell lines were able to internalize both kappa and lambda FLCs at different time points (1 hour (h), 2 h, 6 h, 16 h) but with significant differences: HeLa cells contained less FLCs than the other cell lines after 16 h of incubation. Endothelial and myocardial cells did not reduce their FLCs content throughout 16 h and also seemed to reach a steady state level of paraprotein uptake of both kappa and lambda FLCs. Interestingly FLCs of patients A1k and A2λ are internalized faster than M1k, M2λ and MGUS FLCs in all cell lines particularly in H9C2 cells (Figure 1B). Myocardial cells are probably more rapid in FLCs uptake, needed for proper paraprotein processing and heart protection, in the presence of potential amyloidogenic proteins. Healthy patients (c), with a normal FLCs value and kappa/lambda ratio, were used as controls and they did not show any internalization signal in Western blot (Figure 1A). As shown in Table 1, only the M2λ patient has a single FLCs monoclonal component. Thus to exclude the internalization of intact immunoglobulin complexes we tested two serum sample with a monoclonal component IgG k and IgA λ respectively, combined with normal levels of both kappa and lambda FLCs. As shown in Figure S2 we could not see any kappa or lambda signal under both reducing (S2A) and non reducing (S2B) conditions, indicating that an intact immunoglobulin cannot be internalized in cells under our experimental conditions.

### FLCs are Rerouted in Extracellular Vesicles and Efficiently Re-internalized in Contiguous Cells

Misfolded proteins are able to induce tissue damage via protein fibrillation like the Amyloid Precursor Protein (APP) in Alzheimer Disease. Extracellular protein moieties could be cleared by the microglia or internalized into neurons where they might serve as seeds to induce protein aggregation in neurodegenerative disorders [23].

Misfolded proteins could also be transported via tunnelling nanotubes between cells, within exosomes/microvesicles (EMVs/MVs) or by unconventional secretion of free proteins [30].

**Figure 2:**
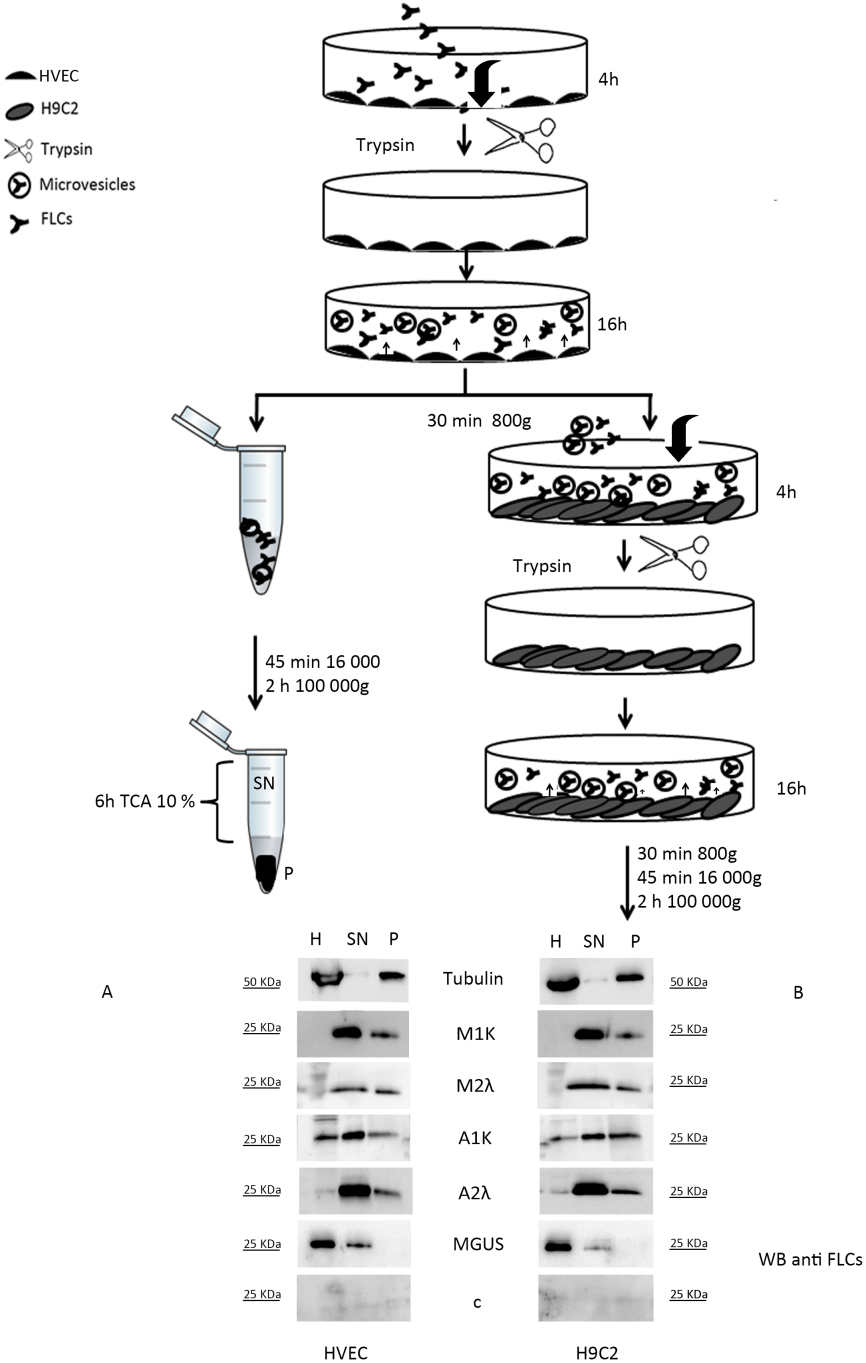
Extracellular transport of internalized FLCs. (A) 7×10^5^ HVEC cells in starvation medium were incubated with serum from M1k, M2λ, A1k, A2λ, MGUS or healthy donor (c) for 4 h. Serum was diluted in starvation medium to obtain 20 µg/mL as final FLCs concentration for all samples. The cells were then washed with PBS 1X, treated with trypsin 170 U 10 min at 4°C and left in fresh starvation medium. After 16 h, starvation medium was collected from HVEC cells, (Microvesicles purification, Methods). Cell extracts (homogenate, H, 30 µg), supernatants (SN, 25 µL) and pellets (P, 25 µL) were run in a native gel 12.5% acrylamide–bisacrylamide gel. Western Blot were performed with anti FLCs and anti tubulin antibodies. (B) HVEC starvation medium obtained after 16 h of chase time was centrifuged at 800×g 30 min. The supernatant was added to H9C2 cell layers in starvation medium for 4 h. The cells were then washed with PBS 1X treated with trypsin 170 U 10 min at 4°C and left in fresh starvation medium. After 16 h of incubation the medium from H9C2 was collected and centrifuged as described in Microvesicles purification, Methods. H, SN and P were run in a native 12.5% acrylamide–bisacrylamide gel and analysed by WB with anti FLCs and anti tubulin antibodies.

We postulated that FLCs might behave in a similar way. Thus we established an experimental protocol to test microvesicles release in HVEC cells. After 4 h of FLCs internalization in endothelial cells and 16 h chase time, cell medium has been collected and analysed for its FLCs content. We adopted a “three steps centrifugation” protocol (see “Materials and Methods”) to eliminate cellular debris, apoptotic membrane patches, etc… The pellets were also filtered on a 0.1 µm mesh to confirm the potential EMVs/MVs content. Surprisingly we could see that internalized FLCs are redirected in the cell culture medium in a soluble (SN) or pellettable (P) form. We postulated that the pellets are composed by EMVs/MVs (Figure 2). In addition we could show that M1k, M2λ, A1k and A2λ FLCs are efficiently delivered in both extracellular spaces (Figure 2A, lane SN and P). M1k and M2λ FLCs are not present in the homogenate (H) after 4 h of internalization and 16 h of chase time showing that all the FLCs that have been internalized were rerouted in the medium and a very little amount is retained in the cells. On the contrary, the MGUS derived FLCs, clearly behave in a different way: the HVEC cells homogenate (H) still contains a significant amount of FLCs (Figure 2A first lane) and only soluble FLCs are released in the culture medium (SN) while there are no FLCs in the pelletable form (P). These data indicate a probably different processing way of some FLCs. Under our experimental conditions, only malignant plasmacells dyscrasia derived FLCs can be secreted in a pelletable form. Cells incubated with healthy donor serum, used as negative control, did not give any signal. To further test a prion like spreading, as suggested in the literature for the APP [31], we incubated myocardial cell lines (H9C2) with the endothelial cells culture medium that holds secreted FLCs and secreted FLCs containing vesicles (see protocol flow chart in Figure 2). We observed an efficient intake of FLCs that where released from endothelial cells (Figure 2B). A more efficient M1k and A2λ FLCs extracellular transport was observed in myoblasts (Figure 2B, SN and P) after co-incubation with endothelial secreted FLC. H9C2 cells maintained the ability to secrete FLCs in the external medium via pelletable (vesicular) structures (P) from M1k, M2λ, A1k, A2λ, but not MGUS patients. Similarly, the healthy donor serum did not give any signal. To confirm that the EMVs/MVs were efficiently internalized, we incubated myoblasts with the pelletted and filtered vesicles, obtained from the endothelial culture medium, and we observed the same result (data not shown).

### Internalized FLCs are Secreted in C-src, Annexin V, Hsp70 Positive Microvesicles

**Figure 3:**
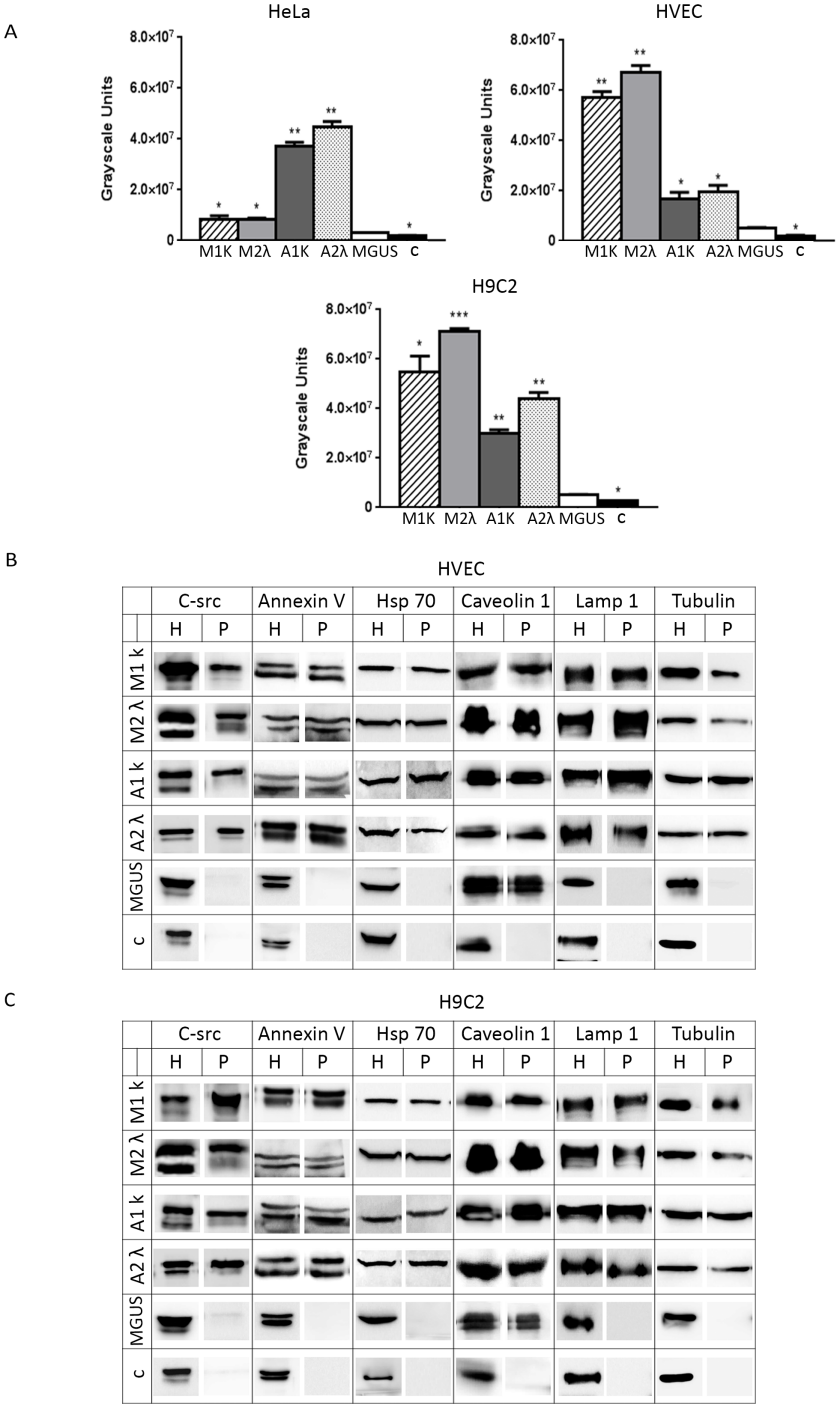
Microvescicle characterization. (A) 7×10^5^ HeLa**,** HVEC or H9C2 cells in starvation medium were incubated with serum from MM (M1k, M2λ), AL amyloidosis (A1k, A2λ), MGUS and healthy donor (c) patients for 4 hours at 37°C. The serum was diluted in starvation medium to a final FLCs concentration of 20 µg/mL for all samples. Cells were then washed with PBS 1X and treated with trypsin as described in Methods and left in fresh starvation medium for 16 h. Starvation medium was harvested, centrifuged at 800×g for 30 min, 16 000×g for 45 min and finally ultracentrifuged at 100 000×g for 2 h (see Microvesicles purification, Methods). WB analysis of cell extracts (homogenate, H, 30 µg) and pellets (P, 25 µL) were performed with anti FLCs antibodies. Figure shows pellets quantification. Results are the mean ±SEM of at least 3 separate experiments. * P<0.05, ** P<0.01 and *** P<0.001. (B,C) HVEC and H9C2 cells were treated as described above. MGUS sample 2.8×10^6^ cells were treated as described. WB was performed with different vesicles markers (c-src, annexinV, Hsp70, caveolin1 and Lamp1) under reducing conditions. H (Homogenate, 30 µg), P (Pellet, 25 µL).

The detection of pellettable FLCs in the culture medium of FLCs pre-treated cells suggested us to compare the vesicular paraprotein release in different cell lines and with different FLCs. As shown in Figure 3A, HeLa cells release a minimum of M1k, M2λ and MGUS derived FLCs instead of A1k and A2λ, showing an alternative way of processing. In endothelial and myocardial cells we observed almost the opposite behaviour: plenty of M1k and M2λ FLCs are secreted in a vesicular form with minor release of A1k and A2λ and minimal release of MGUS FLCs. Cells incubated with healthy donor serum (c) did not give any signal. All results were estimated significant in relation to the MGUS values. (* P<0.05, ** P<0.01 and *** P<0.001). These data indicate that the machinery involved in FLCs processing is cellular and paraprotein type dependent.

We then investigated the nature of microvesicles secreted in the culture medium with different exosomal markers and c-src; c-src has been linked to the inflammatory environment in the myeloma kidney [21] and to the development of cancer [32]. We analysed vesicles produced by HVEC (Figure 3B) and H9C2 (Figure 3C) cells after FLCs internalization. As shown in Figures 3B and 3C we observed the striking presence of the c-src tyrosine kinase on vesicular population (P) generated in both endothelial and myocardial cell lines incubated with M1k, M2λ, A1k and A2λ sera. Furthermore in these samples we revealed the presence of annexin V and the Heat Shock Protein (Hsp)70: two known exosomal markers [33], [34]. In addition, the secreted vesicles harbour the Lysosomal Associated Membrane Protein 1 (Lamp-1) and the integral membrane protein caveolin1 also known to be present in exosomes [34], [35]. In control sample (c) we saw similar signals (Figure 3B) in the homogenate (H) but not in the pelleted fractions (P). Under our experimental conditions in MGUS treated cells we observed a microvesicular population (P), caveolin1 positive, loading 4 times the amount of MM and AL treated cells.

### C-src Intracellular Redistribution After Pathologic FLCs Exposure

**Figure 4:**
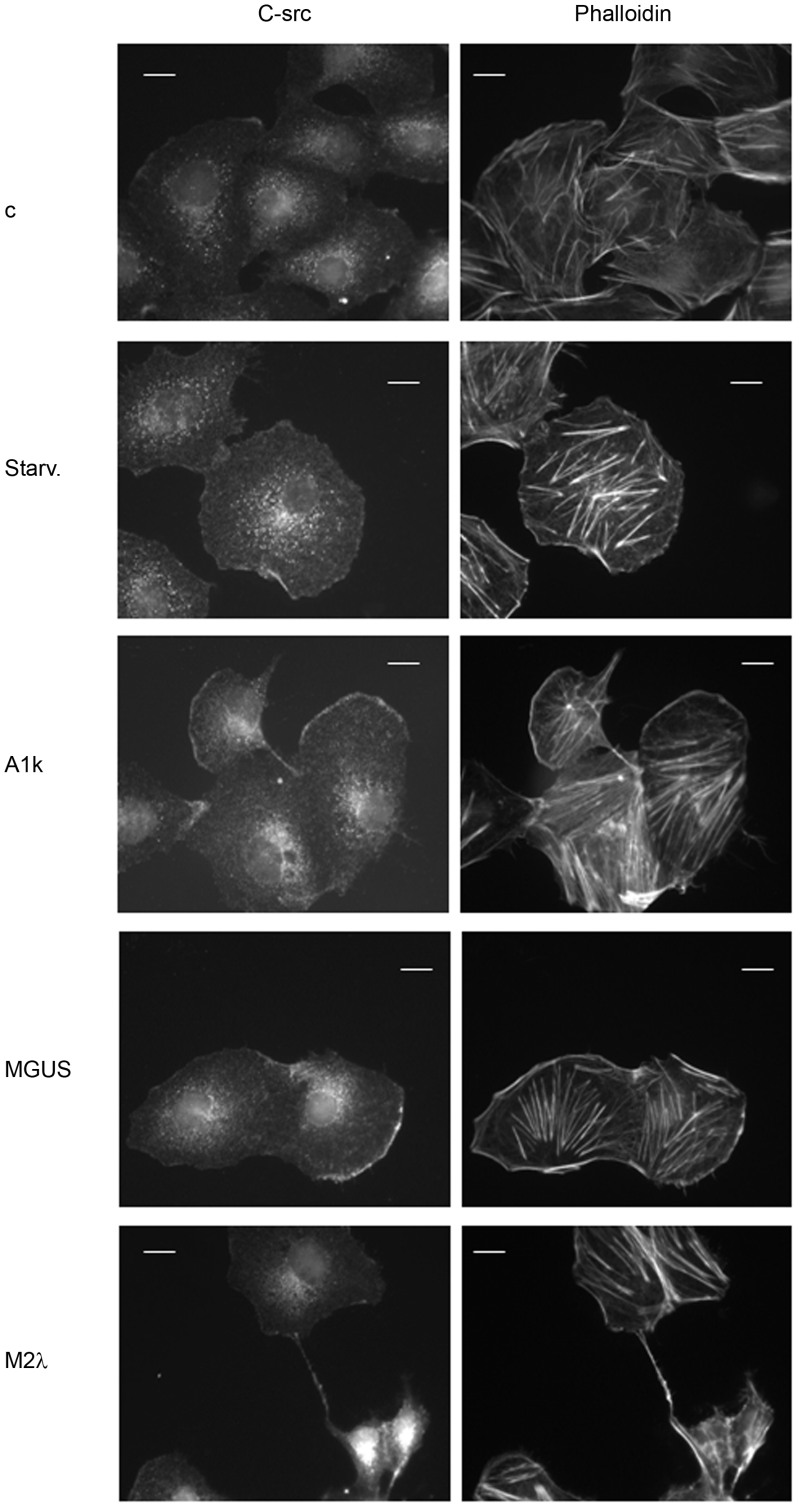
C-src expression and distribution in HVEC cells. HVEC cells were incubated with serum of different patients (healthy patient (c), A1k, MGUS and M2λ) to a final FLCs concentration of 20 µg/mL or with starvation medium (Starv.) for 4 h at 37°C. Cells were fixed with 3% PFA, permeabilized with 0.3% saponin and stained with the c-src antibody, followed by Alexa 488-conjugated anti-rabbit immunoglobulin (Ig), Phalloidin 555 (Alexa Fluor) and DAPI. Scale bars, 5 µm. Coverslips were mounted using an anti-fade mounting medium (ProLong Gold-Invitrogen) on a glass slide. Confocal microscopy was performed on a ZEISS Axiovert 100 fluorescent microscope using the 63× Zeiss oil immersion objective. Single sections are shown for each condition. Images were processed with the use of Image pro-plus 4.5.1.

We saw that FLCs are internalized in HeLa, H9C2 and HVEC cells and they are secreted in c-src positive microvescicles. Previous studies demonstrated that c-src increases its activity in human proximal tubular epithelial cells after urine FLC exposure, showing their ability to generate a proinflammatory environment [20]. Thus we analysed HVEC cells by immunofluorescence after a 4 h incubation with or without serum from all patients (M1k andA2λ gave the same results, respectively, as did M2λ and A1k, data not shown). We observed a clear c-src intracellular re-distribution in cells treated with serum containing FLCs. Figure 4 shows the perinuclear distribution of c-src in cells treated with control serum (c) and untreated cells (Starv). Instead, in MGUS and A1k treated cells, c-src shows a perinuclear distribution and a plasma membrane clustering besides the normal perinuclear distribution. Moreover cells treated with M2λ show an active intercellular communication, with c-src positive tunnels and filopodia structures from cell to cell that we could never observe in MGUS and A1k treated cells. The presence of c-src clusters at plasma membrane in both MGUS and A1k treated cells suggests that c-src is involved in MGUS FLCs cellular processing but not in their vesicle delivery. It will be interesting to monitor this patient in the next years to follow MM progression. Both c-src membrane patches and intracellular tunnels are phalloidin positive, suggesting that microtubules are involved in c-src redistribution. Unfortunately, no commercial anti FLCs antibodies are suitable for immunofluorescence analysis to confirm the presence of FLCs in c-src-phalloidin positive structures. Mineo et al. recently demonstrated that c-src signaling is activated in HUVEC after treatment with exosomes derived from chronic myeloid leukemia cells [36]. They show that large nanotubules containing actin and tubulin modulate exosomes internalization and secretion. These findings support our hypothesis that FLCs exposure activate c-src redistribution in HVEC cells from the perinuclear area to membrane clusters and that, probably, only pathologic FLCs (A1k and M2λ), but not MGUS FLCs, are carried via microvesicles from cell to cell.

### Serum-derived Microvesicles Characterization

**Figure 5:**
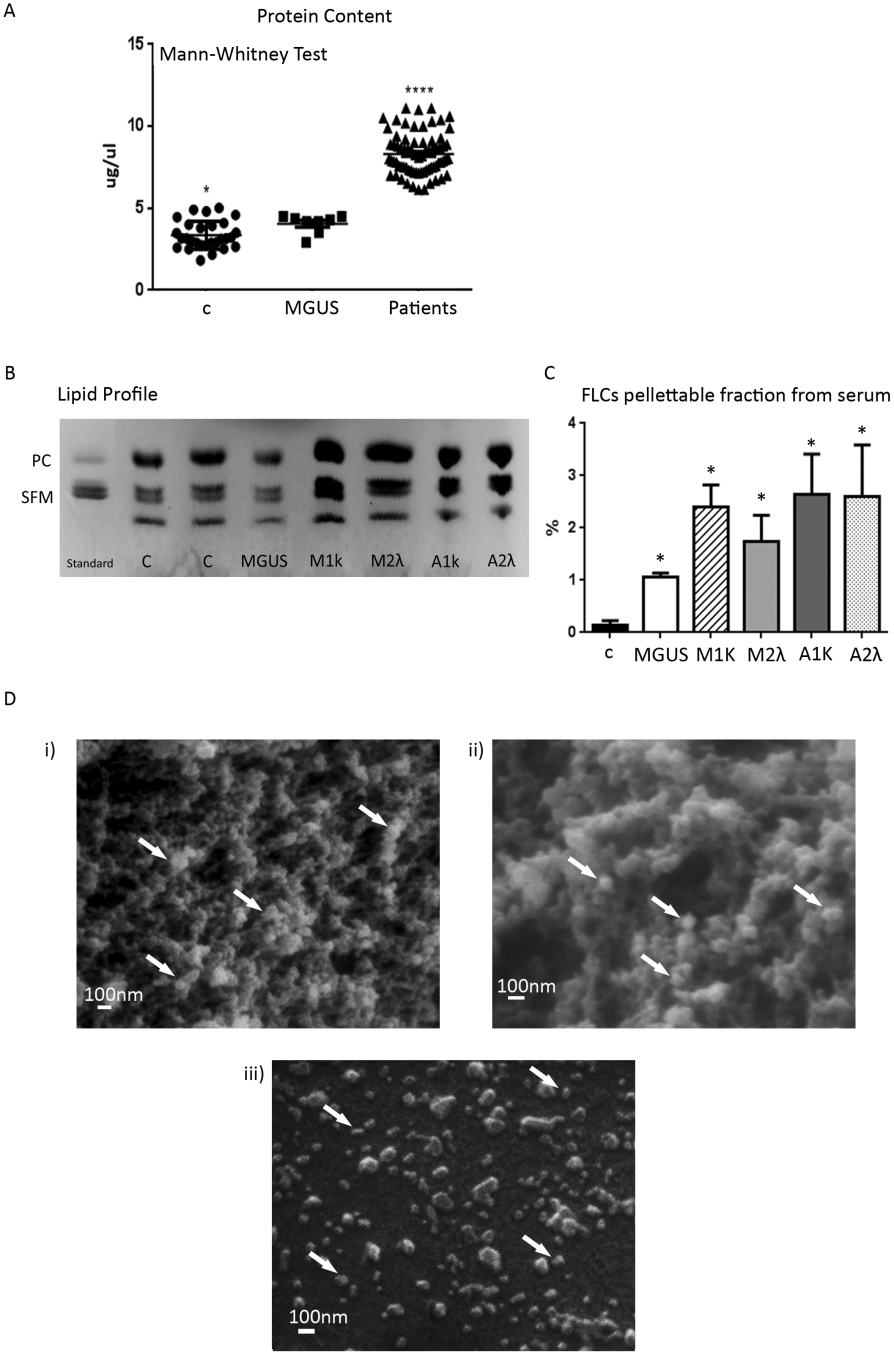
In serum vesicles detection. (A) Microvesicles were purified from 500 µL serum of 3 different patients’ groups: Controls (28 healthy donors), 8 MGUS, Patients (72 malignant plasma cell dyscrasia). Protein content was quantified using Bradford assay. Protocol described in “Vesicles quantification” Methods. Each dot represents each sample quantification, lines are mean ±SEM. Results were analysed using Mann-Whitney test. * P<0.05, **** *P*<0.0001. (B) Microvesicles lipid composition of 1 mL serum from healthy donors (c), MGUS and 500 µL serum from MM (M1k and M2λ) and AL amyloidosis (A1k and A2λ) was detected with Thin Layer Chromatography (PC: phosphatidylcholine, SFM: sphingomyelin). (C) The figure shows the percentage of the FLCs in the pellettable fraction calculated on the total amount of FLCs present in the serum (pellet vs total serum). Results are the mean ±SEM of 2 separate experiments. * P<0.05. (D) Serum pellets obtained with serial centrifugation from M1k patients were fixed in 2,5% glutaraldehyde, dehydrated with acetone, dried in a critical point drying device and gold coated (i 20 000X, ii 40 000 X). Pellets were re-suspended in PBS 1X and centrifuged with a CytoSpin4 for 10 min (iii, 20 000X). Pellets were dried and processes as described above. All samples were visualized with a Philips 501 microscope. White arrows show small vesicles of 60–100 nm in diameter.

To verify the *in vivo* generation of extracellular vesicles we collected serum samples from 72 AL Amyloidosis and MM patients, 8 MGUS and 28 healthy donors (controls). The serum protein profiling data for MM and AL patients are depicted in the (Fig. S3A and Table S1). We quantified the total protein content of the pellets obtained with a “three steps centrifugation” protocol and we plotted the data with the Mann-Whitney Test. As shown in Figure 5A serum samples from malignant plasma cell dyscrasia (Patients) contain a significant (*P*<0.0001) higher amount of pellettable proteins than MGUS and controls (c). Moreover we could show that the serum pellet (P1−) contains FLCs and that most of FLCs are solubilised (SN3+) while few FLCs are left in the pelletable form (P3+) after Triton-X 100 treatment (Figure S3B). We can also conclude that FLCs are present in the inner side of the vesicular population because of trypsin pre-treatment (P1−) (see Methods).

**Figure 6:**
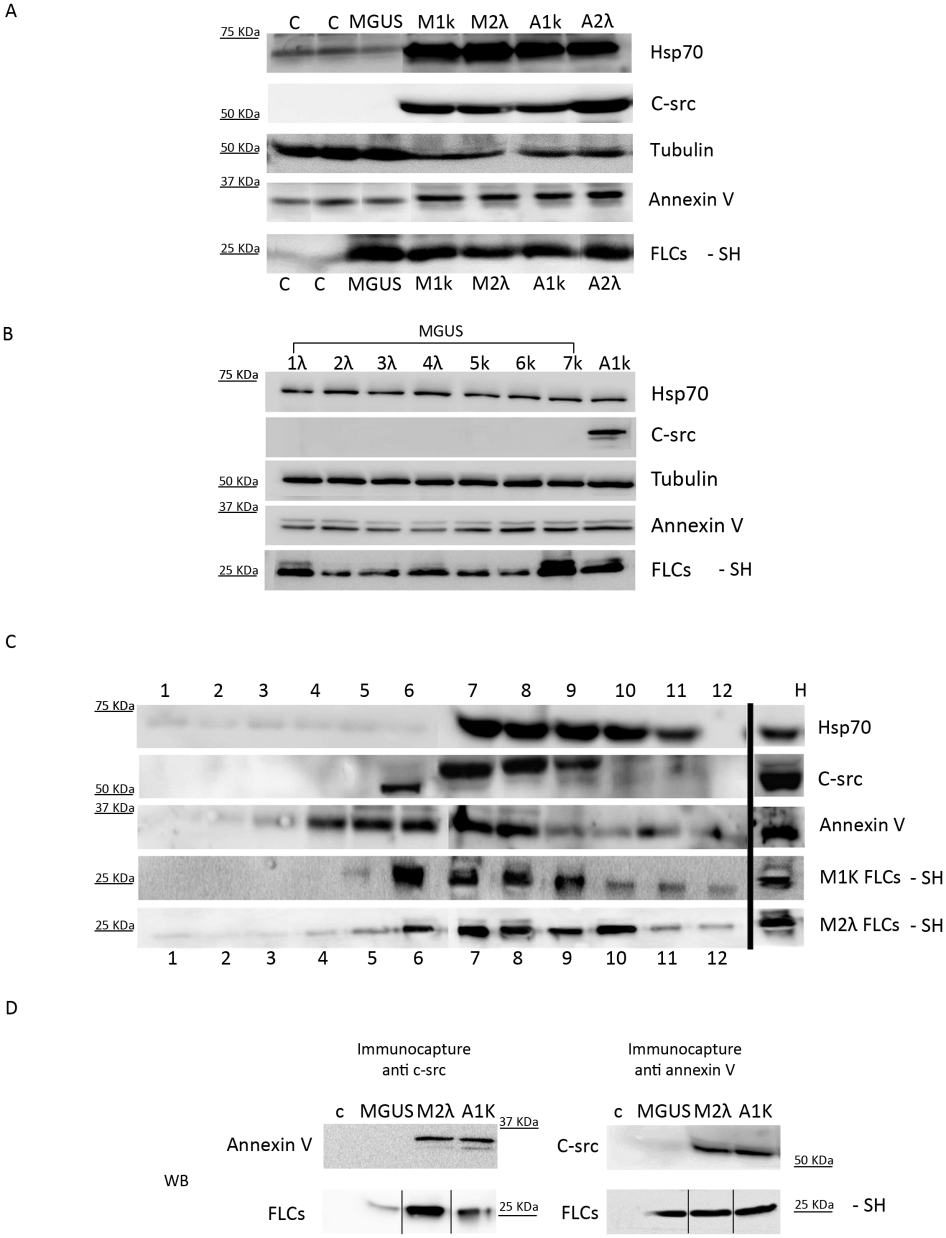
In serum vesicles characterization. (A, B) Microvesicles purified from 500 µL serum of healthy donors (c), 8 MGUS, MM (M1K, M2λ) and AL amyloidosis (A1k, A2λ) were analysed by WB with different vesicles markers. (C) Microvesicle pellets were loaded on the top of a 15–60% discontinuous sucrose gradient. Twelve fractions of equal volume were collected from the top (low density, fraction 1) to the bottom (high density, fraction 12). Samples were electrophoresed and analysed by WB using anti Hsp70, c-src, annexin V, kappa and lambda FLCs antibodies. (D) Micro-vesicles purified from serum of healthy donors (c), MGUS, M2λ A1k were loaded on the discontinuous sucrose gradient. Fractions from 6 to 9 were incubated with magnetic beads coupled with anti c-src or annexin V antibodies for 16 h. Beads were captured with a magnet, washed with PBS 1X and re-supended in 50 µL of reducing or not reducing sample buffer and heated at 95°C for 5 min. Samples were run in SDS-PAGE and native conditions and analysed by WB using anti annexin V, c-src and FLCs antibodies.

We analysed the lipid composition of the microvesicles/exosomes population of M1k, M1λ, A1k, A2λ, and MGUS patients and two healthy donors (c). The purified serum pellets were loaded on a thin layer cromatography (TLC) system: all samples contained Phosphatidylcholine (PC) and Sphingomyelin (SFM) (Figure 5B). For the controls (c) and MGUS we loaded twice the protein concentration of MM and AL pellets to reveal their lipids content, further confirming that an increase of extracellular vesicles occurs in MM and AL. To test the pellettable amount of FLCs in serum samples we processed M1k, M2λ, A1k, A2λ, MGUS and control pellets under native conditions. Figure 5C shows that the percentage of the FLCs in the pellettable fraction is around 2% of the FLCs present in the patients’ serum. These results are significant (*P<0.05) compared to the FLCs pelletable fraction of healthy donorsTo better characterize the composition of serum pelleted material we performed morphological analyses using SEM. Figure 5D shows the presence of vesicles with diameters from 60 to 100 nm in M1k serum patients. The pelleted serum derived vesicles were observed as clusters of vesicles (Figure 5D i, ii) or single vesicles (Figure 5D, iii), Similar morphology was described for exosomes isolated from different biological fluids [37]. The vesicles content was further characterized by determining the presence of Hsp 70, annexin V, tubulin and c-src. Our data (Figure 6A) clearly show that plasma derived microvesicles from MM (M1k and M2λ) and Amyloidosis AL (A1k and A2λ) patients contain FLCs and express high levels of Hsp 70, annexin V and c-src. Healthy donors (c) and MGUS derived samples present exosomal associated proteins like annexin V, Hsp70 and tubulin. However, c-src was almost absent from these samples. These data have been confirmed for all the 8 MGUS patients analysed (Figure 6B). To confirm that a microvesicles/exosomes population contains FLCs, we performed a sucrose gradient protocol. As shown in Figure 6C, fractions 7 and 8 contained the FLCs (both kappa and lambda) and are Hsp70, c-src and annexin V positive, We show only A1k and M2λ, because the other patients (M1k and A2λ) gave the same results.

Finally to confirm that FLCs are present in c-src and annexin V positive vesicles we performed an immunocapture assay: fractions 6 to 9 from the discontinuous gradient were collected and incubated with c-src or annexin V coupled magnetic beads. As figure 6D shows, the c-src positive vesicles from M2λ and A1k, but not from MGUS patients and healthy donor (c), contain annexin V and FLCs. Samples immunocaptured with anti annexin V antibody show a FLCs signal in MGUS, M2λ and A1k, but not in the control. Moreover, c-src is visible only in M2λ and A1k, but not in MGUS and c. Thus only FLCs produced during malignant plasma cells dyscrasia are transported via c-src and annexin V positive EMVs/MVs.

## Discussion

In the present study we demonstrated that serum FLC are efficiently internalized in endothelial and myocardial cell lines. Furthermore we revealed a novel mechanism for FLCs processing that involves c-src labelled extracellular vesicles. Our *in vitro* data are strongly confirmed by the characteristics of microvesicles produced *in vivo*. More strikingly MGUS derived microvesicles are different from those produced in MM or AL Amyloidosis, suggesting a new prognostic tool.

Plasma cell dyscrasia is characterized by proliferation of uncontrolled plasma cells and the accumulation of their secreted paraproteins. Currently, patients with a malignant form (MM) of plasma cell dyscrasia have a short life expectancy. Organs including the lungs and heart can also be affected; if AL Amyloidosis affects the myocardium, it could potentially cause of morbidity.

More than one trigger factor could be involved in the prognosis and progression of MM, including: kidney failure, AL Amyloidosis with heart deficiency, LCDD, tubular cast nephropathy, osteolitic lesions, peripheral neuropathy and immunodeficiency. Each patient shows his own history and to date it is quite hard to predict if MGUS paraproteins will be able to damage tissues.

LCDD and AL Amylodosis have been linked to different intracellular trafficking pathways in mesangial cells: amyloidosis light chains (AL-LCs) are delivered to the mature lysosomal compartment where amyloid formation occurs and LCDD-LCs alter mesangial function and phenotype [20]. To investigate the biological effect of FLCs in the blood stream and the underlying molecular mechanism, we analysed the processing of paraproteins in endothelial and myocardial cells before kidney ultrafiltration. The patient’s group we selected for endocytosis assays included only one patient with FLC lambda Myeloma (M2λ) and 4 subjects (M1k, A1k, A2λ and MGUS) with complete immunoglobulin component associated with a FLCs component. In our preliminary experiments we also tested two serum samples with complete monoclonal immunoglobulin (IgGk and IgAλ) with normal FLCs values. In these experiments we could not see any FLCs internalization signal, under non reducing conditions, in endothelial and myocardial cell lines. In contrast we observed an efficient intake of FLCs in both endothelial and myocardial cells for all patients with in excess of FLCs. These data indicate that, under our experimental conditions, monoclonal FLCs can be internalized in myocardial and endothelial cells. The whole immunoglobulin complex, if recycled through the neonatal Fc receptor, was not detectable in the cell homogenates that we analyzed. We also observed faster internalization rates for A1k and A2λ in the two patients with AL Amyloidosis, compared to the other patients. Thus we confirmed a higher avidity for AL-LCs uptake in the vascular bed, such as the one present in mesangial cells [20]. Different turnovers are probably due to disposal of proteins either less or more toxic. Proteins could be potent messengers that activate trafficking pathways in different cell types. Like the Aβ peptide, a component of amyloid plaques in Alzheimer disease, paraproteins could probably trigger proinflammatory events. We hypothesized a potential prion like propagation [38], [39] from endothelial to myocardial cell lines. The neurodegeneration has been linked to an exosomal shipping of proteins [31]. Exosomal release might be of advantage to cells having poor degradation capacities: Initially, it would be beneficial to cells but later on it would become dangerous. In order to demonstrate this hypothesis we checked for EMVs/MVs production of human cell lines after serum containing high amounts of FLCs exposure. Our data showed that FLCs are redirected in the extracellular space in a soluble form and in vesicular structures. The recent study on urinary exosomes containing large amounts of free immunoglobulin light chains, were isolated from AL Amyloidosis and MM urine samples corroborates our findings [40]. Moreover we observed the striking evidence that MGUS FLCs behave differently: the internalized FLCs are transported in the culture medium mainly in a soluble form. In addition we could show that endothelial secreted EMVs/MVs are able to transfer their FLCs content into myocardial cells thus suggesting a FLCs shipping process.

Macrophage-produced MVs bind to adjacent cells, including macrophages and dendritic cells, leading to their activation and resulting in secretion of TNF-α and potentially other proinflammatory mediators [41]. Sterile inflammation can be a major factor that triggers vascular damage and subsequent fibrosis [42]. Receptor-mediated endocytosis and metabolism of monoclonal FLCs generates an intrarenal proinflammatory environment that exacerbates ongoing renal injury and tubulointerstitial fibrosis in a NF-kB mediated way [43]. It has been shown that immunoglobulin FLCs activate both the canonical and atypical (IKK-independent) NF-kB pathways through a c-src tyrosine phosphorylation [21]. Our observation of c-src recruitment on microvesicles, which contain immunoglobulin light chains, suggest a novel mechanism of c-src activation during plasma cell dyscrasia and its potential involvement in proinflammatory events and tissue damage. The evidence of plasma membrane recruitment of c-src and intercellular tunneling of c-src positive carriers after FLCs exposure, represents a possible mechanisms linked to the paraprotein pathogenicity.

It would be of great interest to analyse serum samples in different MGUS patients before and after switching in malignant plasma cell dyscrasia. The striking evidence that the MGUS derived FLCs are unable to induce the c-src enrichment on secreted microvesicles *in vivo* and *in vitro* requires the study of a larger group of patients. If confirmed, the quantification of c-src positive microvesicles could be a new diagnostic tool for the pathogenicity prediction of paraproteins.

All these data concerning the cellular metabolism of paraproteins highlight the need to identify the behaviour of different serum MVs produced by different patients. Further microvesicle and exosomes characterization of MM versus control patients will surely improve our knowledge at the molecular level and provide a novel tool in diagnosing plasma cell dyscrasia patients.

## Supporting Information

Figure S1 **Trypsin treatment, after internalization, efficiently eliminates all type of residual FLCs from cell membrane. (**A) HVEC cells in starvation medium were incubated with serum containing FLCs from M2λ patient for 4 h at 37°C. The serum was diluted directly in starvation medium to a final FLCs concentration of 20 µg/mL. After incubation cells were washed carefully at 4°C with PBS 1X, incubated 5 min at 4°C and 10 min at 37°C with 170 U trypsin (+) or not (−). Cells were processed as described in “Cytosol/membrane separation” (Methods) (H, homogenate; cyt, cytosol; mb, membrane). 30 ug of each sample were loaded on a SDS–PAGE or native gel and analysed by WB anti FLCs and flotilin, the last used as a membrane marker. (B) HVEC cells were incubated with serum containing FLCs from MM (M1k, M2λ), AL amyloidosis (A1K, A2λ), MGUS or healthy donor (c) for 4 h. Serum was diluted to obtain 20 µg/mL as final FLCs concentration for all samples. Cells were then treated with trypsin (+), as described above, and processed for the Cytosol/membrane separation (H, homogenate; mb, membrane). 30 ug of each sample were loaded on a SDS-PAGE or a native gel and analysed as described.
(TIF)

Figure S2 **Denaturing or native electrophoresis analysis of internalized FLCs. (**A) HVEC cells in starvation medium were incubated with serum containing FLCs from AL amyloidosis (A1k, A2λ), two patients with monoclonal component but normal FLCs levels (IgGk and IgAλ) or healthy donor patients (c) for 4 h at 37°C. The serum was diluted in starvation media to a final FLCs concentration of 20 µg/mL for all samples. After incubation cells were washed carefully at 4°C with PBS 1X, incubated 5 min at 4°C and 10 min at 37°C with 170 U of trypsin to remove any FLCs attached to cell membrane and processed as described in “ Internalization assay” (Methods). Samples were resuspended in SDS sample buffer. 30 µg of each sample were run in SDS-PAGE, 12.5% acrylamide–bisacrylamide gel. B) HVEC cells were processed as reported above and the samples were resuspended in non-reducing SDS-free sample buffer. 30 µg of each sample were run in a native 12.5% acrylamide–bisacrylamide gel. Western Blot (WB) analysis was performed with anti kappa, anti lambda FLCs and anti tubulin antibodies
(TIF)

Figure S3 **Characterization of patients’ sera.** (A) Percentage of monoclonal component isotypes:. each column represent the percentage of patients producing the same monoclonal component isotype. “Others”, is a smaller group with: double IgGk, k, λ, IgAλ+ IgGk, IgGk+λ, IgMk, IgMk+IgMλ, IgMλ+IgGk. The mean value of kappa and lambda FLCs concentrations in all patients is also shown. B) 500 µL of M2λ patient serum were processed for microvesicles purification with serial ultracentrifugation steps as described in Methods (Vesicles lysis) and processed with Triton-X 100 to confirm that FLCs were in the vesicles. WB was performed with anti lambda FLCs antibodies. Figure shows that the untreated serum pellet (P1) contained FLCs, but after Trypsin,Triton-X 100 treatment, and centrifugation, the majority of FLCs were in the supernatant (SN3+) and not in the pellettable form (P3+) (Figure S3B).
(TIF)

Table S1 **Characterization of the monoclonal components of all patients involved in this study.** The table shows: serum immunofixation (IF-S) and urine immunofixation (IF-U) results; kappa and lambda FLCs serum concentration and ratio; immunoglobulin quantification (IgA- IgG- IgM). N.a.: not available.
(DOCX)
