# Supplementary figures and images for: C-src Enriched Serum Microvesicles Are Generated in Malignant Plasma Cell Dyscrasia
Source: PLoS One. 2013 Aug 5;8(8):e70811. doi: 10.1371/journal.pone.0070811 (PMC3733647; doi:10.1371/journal.pone.0070811)

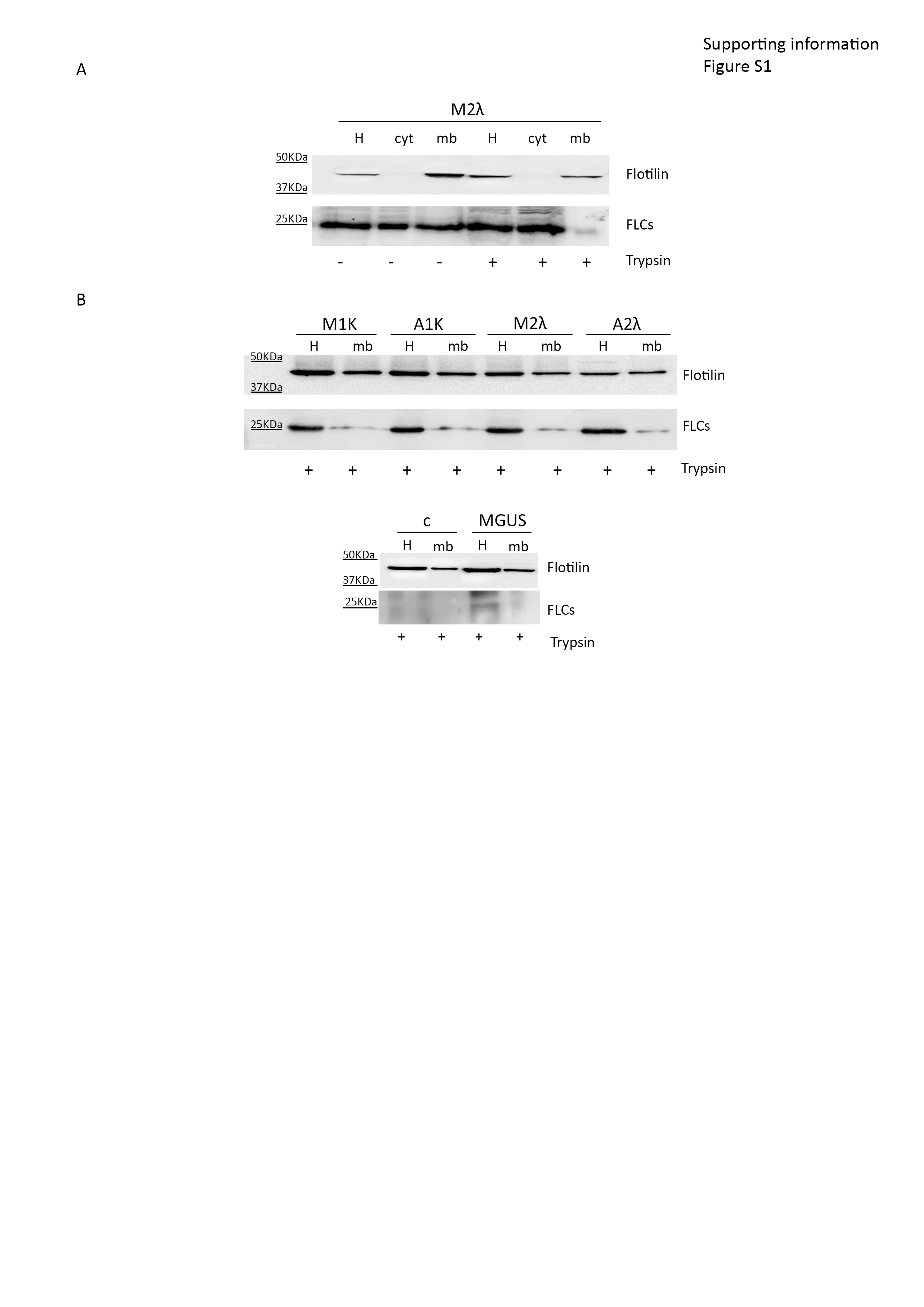

Supplement: Figure S1 — Trypsin treatment, after internalization, efficiently eliminates all type of residual FLCs from cell membrane. (A) HVEC cells in starvation medium were incubated with serum containing FLCs from M2λ patient for 4 h at 37°C. The serum was diluted directly in starvation medium to a final FLCs concentration of 20 µg/mL. After incubation cells were washed carefully at 4°C with PBS 1X, incubated 5 min at 4°C and 10 min at 37°C with 170 U trypsin (+) or not (−). Cells were processed as described in “Cytosol/membrane separation” (Methods) (H, homogenate; cyt, cytosol; mb, membrane). 30 ug of each sample were loaded on a SDS–PAGE or native gel and analysed by WB anti FLCs and flotilin, the last used as a membrane marker. (B) HVEC cells were incubated with serum containing FLCs from MM (M1k, M2λ), AL amyloidosis (A1K, A2λ), MGUS or healthy donor (c) for 4 h. Serum was diluted to obtain 20 µg/mL as final FLCs concentration for all samples. Cells were then treated with trypsin (+), as described above, and processed for the Cytosol/membrane separation (H, homogenate; mb, membrane). 30 ug of each sample were loaded on a SDS-PAGE or a native gel and analysed as described. (TIF) [file pone.0070811.s001.tif]

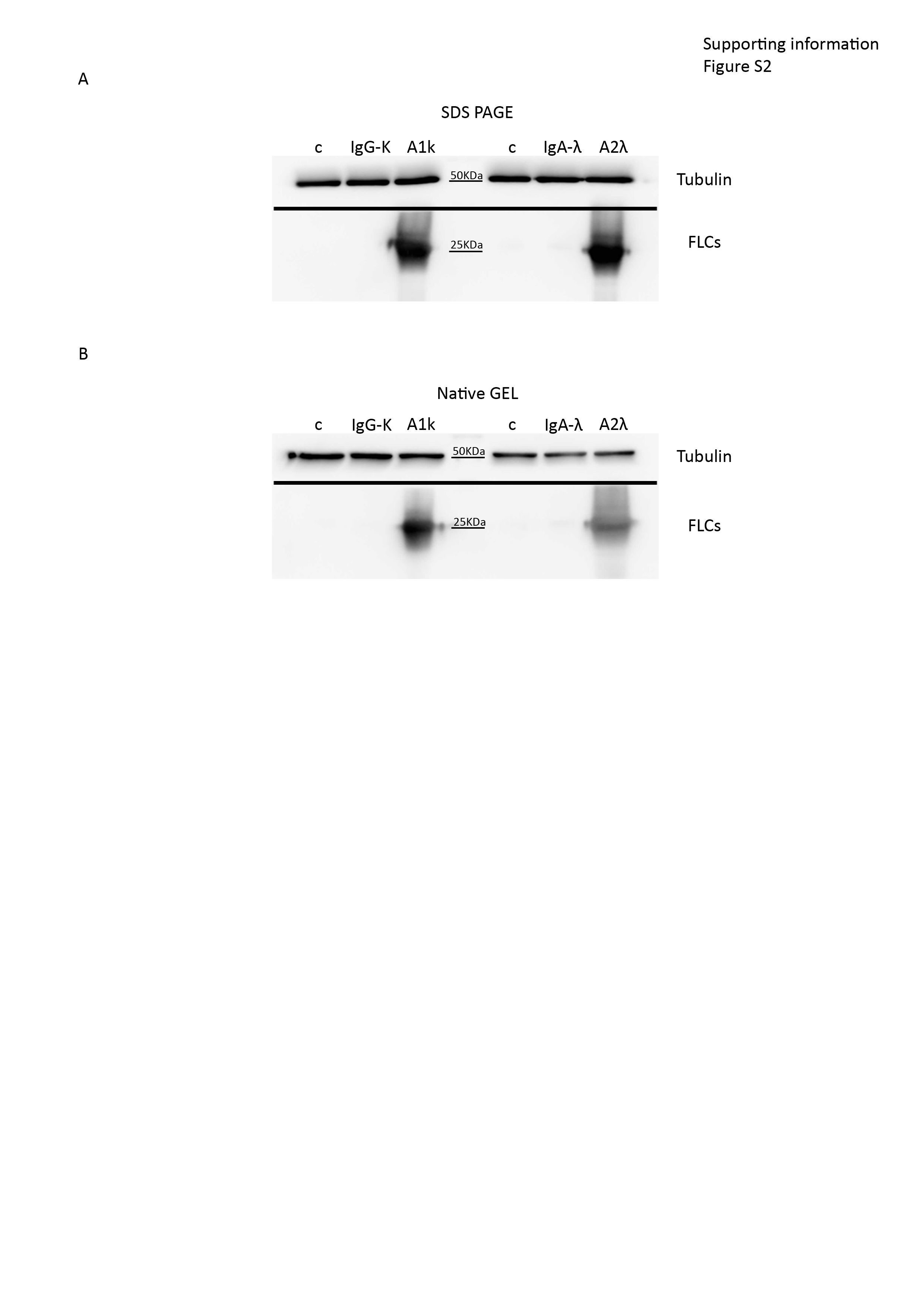

Supplement: Figure S2 — Denaturing or native electrophoresis analysis of internalized FLCs. (A) HVEC cells in starvation medium were incubated with serum containing FLCs from AL amyloidosis (A1k, A2λ), two patients with monoclonal component but normal FLCs levels (IgGk and IgAλ) or healthy donor patients (c) for 4 h at 37°C. The serum was diluted in starvation media to a final FLCs concentration of 20 µg/mL for all samples. After incubation cells were washed carefully at 4°C with PBS 1X, incubated 5 min at 4°C and 10 min at 37°C with 170 U of trypsin to remove any FLCs attached to cell membrane and processed as described in “ Internalization assay” (Methods). Samples were resuspended in SDS sample buffer. 30 µg of each sample were run in SDS-PAGE, 12.5% acrylamide–bisacrylamide gel. B) HVEC cells were processed as reported above and the samples were resuspended in non-reducing SDS-free sample buffer. 30 µg of each sample were run in a native 12.5% acrylamide–bisacrylamide gel. Western Blot (WB) analysis was performed with anti kappa, anti lambda FLCs and anti tubulin antibodies (TIF) [file pone.0070811.s002.tif]

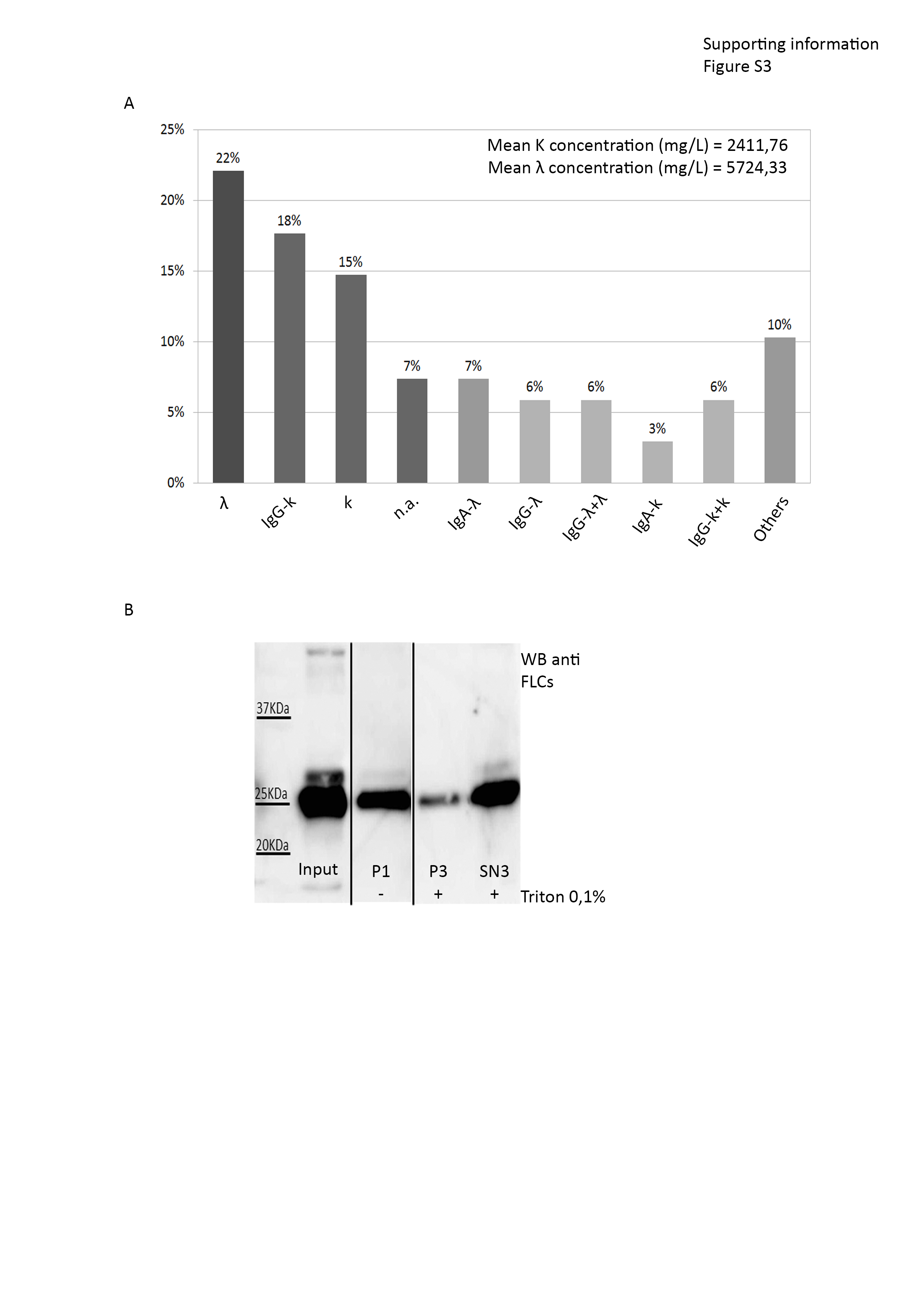

Supplement: Figure S3 — Characterization of patients’ sera. (A) Percentage of monoclonal component isotypes:. each column represent the percentage of patients producing the same monoclonal component isotype. “Others”, is a smaller group with: double IgGk, k, λ, IgAλ+ IgGk, IgGk+λ, IgMk, IgMk+IgMλ, IgMλ+IgGk. The mean value of kappa and lambda FLCs concentrations in all patients is also shown. B) 500 µL of M2λ patient serum were processed for microvesicles purification with serial ultracentrifugation steps as described in Methods (Vesicles lysis) and processed with Triton-X 100 to confirm that FLCs were in the vesicles. WB was performed with anti lambda FLCs antibodies. Figure shows that the untreated serum pellet (P1) contained FLCs, but after Trypsin,Triton-X 100 treatment, and centrifugation, the majority of FLCs were in the supernatant (SN3+) and not in the pellettable form (P3+) (Figure S3B). (TIF) [file pone.0070811.s003.tif]
