# Supplementary material for: C-src Enriched Serum Microvesicles Are Generated in Malignant Plasma Cell Dyscrasia
Source: PLoS One. 2013 Aug 5;8(8):e70811. doi: 10.1371/journal.pone.0070811 (PMC3733647; doi:10.1371/journal.pone.0070811)
Supplement: Table S1 — Characterization of the monoclonal components of all patients involved in this study. The table shows: serum immunofixation (IF-S) and urine immunofixation (IF-U) results; kappa and lambda FLCs serum concentration and ratio; immunoglobulin quantification (IgA- IgG- IgM). N.a.: not available. (DOCX) [file pone.0070811.s004.docx]

**Table S1: Characterization of the monoclonal components of all patients involved in this study.**

| **Patients** | **S- IF** | **U- IF** | **K** | **λ** | **Ratio** | **IgA** | **IgG** | **IgM** |
| --- | --- | --- | --- | --- | --- | --- | --- | --- |
| 1 | K | neg. | 5960 | 7.9 | 754.4304 | 76.2 | 1010 | 18 |
| 2 | K | K | 63800 | 1.26 | 50634.92 | 22.9 | 474 | 10.5 |
| 3 | IgG-K | K | 226 | 0.3 | 753.3333 | 47 | 1203 | 22 |
| 4 | λ | λ | 2.74 | 932 | 0.00294 | 105 | 659 | 51.3 |
| 5 | IgG-K | IgG-K | 3450 | 2.7 | 1277.778 | 179 | 897 | 27 |
| 6 | IgG-K | neg. | 394 | 160 | 2.4625 | 450 | 1010 | 93 |
| 7 | λ | n.a. | 0.42 | 2100 | 0.0002 | 98 | 718 | 26 |
| 8 | IgG-λ | λ | 1.8 | 1690 | 0.001065 | 24 | 420 | 21 |
| 9 | λ | λ | 9.27 | 2200 | 0.004214 | n.a. | n.a. | n.a. |
| 10 | IgA-K | K | 2650 | 0.7 | 3785.714 | 159 | 928 | 103 |
| 11 | n.a. | n.a. | 1.06 | 4680 | 0.000226 | 30 | 1166 | 27 |
| 12 | IgG-K | K | 904 | 0.39 | 2317.949 | 45 | 1420 | 130 |
| 13 | λ | λ | 53 | 1674 | 0.031661 | 33 | 388 | 21 |
| 14 | IgA-λ + IgG-K | neg. | 21 | 840 | 0.025 | 1101 | 829 | 54 |
| 15 | IgG-K | λ | 28 | 300 | 0.093333 | n.a. | n.a. | n.a. |
| 16 | IgG-K | K | 620 | 0.8 | 775 | 63.9 | 1760 | 30.2 |
| 17 | n.a. | λ | 0.34 | 23000 | 1.48E-05 | 8 | 224 | 5 |
| 18 | n.a. | n.a. | 600 | 16.6 | 36.14458 | 498 | 771 | 97 |
| 19 | IgG-λ + λ | IgG-λ + λ | 10.4 | 1230 | 0.008455 | 64.5 | 1880 | 85.6 |
| 20 | IgG-K | λ | 3 | 3080 | 0.000974 | 7.78 | 1270 | 7.57 |
| 21 | IgG-K + K | K | 1260 | 12.8 | 98.4375 | 30 | 2515 | 28 |
| 22 | k | K | 2100 | 40.9 | 51.34474 | 98 | 347 | 10 |
| 23 | Double IgG-K | K | 704 | 4.3 | 163.7209 | 84 | 1152 | 14 |
| 24 | IgG-K | K | 2223 | 0.45 | 4940 | 8 | 248 | 5 |
| 25 | λ | neg. | 0.8 | 1892 | 0.000423 | n.a. | n.a. | n.a. |
| 26 | IgG-λ | λ | 1.89 | 4960 | 0.028836 | 19 | 1620 | 17 |
| 27 | IgG-λ + λ | IgG-λ | 28 | 971 | 0.028836 | 146 | 1240 | 48.4 |
| 28 | IgG-K | IgG-K + K | 1195 | 11.04 | 108.2428 | 60 | 2793 | 33 |
| 29 | λ | λ | 27.2 | 971 | 0.028012 | 47 | 1040 | 58.9 |
| 30 | IgG-K + λ | n.a. | 6.9 | 4820 | 0.001432 | 42 | 712 | 13 |
| 31 | K | neg. | 2030 | 3.7 | 548.6486 | 7.53 | 312 | 7.55 |
| 32 | IgG-K | K | 4920 | 2.21 | 2226.244 | 17 | 507 | 35 |
| 33 | K | K | 10100 | 51.3 | 196.8811 | n.a. | n.a. | n.a. |
| 34 | IgA-λ | n.a. | 158.5 | 646.4 | 0.245204 | 699 | 838 | 42 |
| 35 | IgG-k+k | k | 1960.6 | 76.97 | 25.47226 | 104 | 789 | 44 |
| 36 | K | IgG-K + K | 2420 | 7.88 | 307.1066 | 16.2 | 8030 | 80.81 |
| 37 | IgM-K + IgM-λ | Double λ | 0.28 | 1363 | 0.000205 | 333 | 3470 | 941 |
| 38 | Double λ | λ | 0.09 | 4819 | 1.87E-05 | n.a. | n.a. | n.a. |
| 39 | IgA-λ | neg. | 23.9 | 113 | 0.211504 | 692 | 1151 | 124 |
| 40 | IgG-λ | λ | 100 | 2000 | 0.05 | 52 | 1462 | 32 |
| 41 | IgG-λ + λ | IgG-λ | 1590 | 1540 | 1.032468 | 58 | 1570 | 62 |
| 42 | IgA-λ | λ | 14.2 | 2918 | 0.004866 | 3624 | 738 | 65 |

| 43 | IgG-K | K | 231 | 29.7 | 7.777778 | 117 | 1860 | 22.7 |
| --- | --- | --- | --- | --- | --- | --- | --- | --- |
| 44 | IgM-K | K | 547.6 | 64.3 | 8.51 | 80 | 986 | 66 |
| 45 | IgG-λ + λ | n.a. | 1.34 | 267 | 0.005019 | 42.7 | 3390 | 18.9 |
| 46 | K | K | 1592 | 38.65 | 41.19017 | 211 | 1400 | 23.1 |
| 47 | IgA-K | n.a. | 917 | 5.3 | 173.0189 | 113 | 1110 | 27.6 |
| 48 | IgG-K + K | IgG-K + K | 15600 | 17.9 | 871.5084 | 16 | 1530 | 12 |
| 49 | λ | λ | 21.5 | 514 | 0.041829 | 116 | 795 | 38 |
| 50 | n.a. | K | 1620 | 3.03 | 534.6535 | 31.8 | 5430 | 25.7 |
| 51 | IgG-λ | IgG-λ + λ | 14.4 | 170 | 0.084706 | 15.7 | 5110 | 5 |
| 52 | K | K | 5640 | 30.8 | 183.1169 | 125 | 734 | 17.6 |
| 53 | IgA-λ | IgA-λ | 4.4 | 388 | 0.01134 | 900 | 672 | 22.7 |
| 54 | λ | λ | 6.14 | 648.4 | 0.009469 | 88 | 544 | 13 |
| 55 | λ | λ | 176 | 186 | 0.946237 | 227 | 1312 | 65 |
| 56 | K | K | 1198 | 3.46 | 346.2428 | 51 | 350 | 15 |
| 57 | λ | λ | 0.9 | 2067 | 0.000435 | n.a. | n.a. | n.a. |
| 58 | λ | λ | 7.63 | 2469 | 0.003348 | 67 | 471 | 8 |
| 59 | λ | λ | 3.15 | 941 | 0.003348 | 22.6 | 581 | 23.7 |
| 60 | IgM-λ + IgG-K | λ | 0.5 | 631 | 2540.146 | 26.2 | 743 | 23.7 |
| 61 | K | K | 34800 | 13.7 | 2540.146 | 30 | 585 | 25 |
| 62 | K | K | 5200 | 20.1 | 258.7065 | 19.8 | 170 | 8.22 |
| 63 | λ | λ | 6 | 285 | 0.021053 | 22 | 800 | 47 |
| 64 | IgG-K + K | IgG-K + K | 1960 | 6.57 | 298.3257 | 3 | 5536 | 5 |
| 65 | IgG-K | IgG-K | 118 | 8.3 | 14.21687 | 28 | 383 | 11 |
| 66 | λ | λ | 1 | 11483 | 8.71E-05 | 302 | 907 | 59 |
| 67 | IgA-λ | λ | 2.33 | 246 | 0.009472 | 298 | 1010 | 89 |
| 68 | n.a. | n.a. | 1480 | 63.9 | 23.16119 | n.a. | n.a. | n.a. |
| IgG-k | IgG-k | neg. | 20.1 | 14.5 | 1.38 | 200 | 1802 | 110 |
| IgA-λ | IgA-λ | neg. | 12.6 | 18.9 | 0.66 | 800 | 1010 | 90 |
| MGUS 1λ | IgG-λ | λ | 1.69 | 608 | 0.00278 | 20 | 2010 | 28 |
| MGUS 2λ | IgA-λ | neg | 12.9 | 105.7 | 0.122044 | 719 | 908 | 66 |
| MGUS 3λ | IgG-λ | λ | 9.37 | 284 | 0.032993 | 28.00 | 2220.00 | 15.00 |
| MGUS 4λ | IgG-λ | λ | 21 | 218 | 0.09633 | 180 | 1470 | 83 |
| MGUS 5k | IgG-k | K | 266 | 2.4 | 110.8333 | n.a. | n.a. | n.a. |
| MGUS 6k | IgM-K | K | 159.04 | 9.75 | 16.31179 | 39 | 291 | 5780 |
| MGUS 7k | n.a. | n.a. | 1110 | 58.2 | 19.07216 | n.a. | n.a. | n.a. |
